# Efficacy and Safety of Autologous Stem-Cell Transplantation as Part of First-Line Treatment for Newly Diagnosed Primary Central Nervous System Lymphoma: A Systematic Review and Meta-Analysis

**DOI:** 10.3389/fonc.2021.799721

**Published:** 2022-01-12

**Authors:** Jing Liu, Jiayuan Guo, Xuefei Sun, Yuanbo Liu, Chunji Gao

**Affiliations:** ^1^ Senior Department of Hematology, The Fifth Medical Center of People's Liberation Army (PLA) General Hospital, Beijing, China; ^2^ School of Medicine, Nankai University, Tianjin, China; ^3^ Department of Hematology, Beijing Tiantan Hospital, Capital Medical University, Beijing, China

**Keywords:** primary central nervous system lymphoma, autologous stem-cell transplantation, meta-analysis, prospective studies, whole-brain radiotherapy, relapse

## Abstract

**Objective:**

The reviewed literature supports a treatment regimen for primary central nervous system lymphoma (PCNSL) that includes induction chemotherapy, followed by one consolidation therapy. High-dose chemotherapy supported by autologous stem-cell transplantation (ASCT) is the most studied option, but its effects are controversial. The aim of this study was to evaluate the efficacy and safety of ASCT for newly diagnosed PCNSL by means of a meta-analysis.

**Methods:**

The PubMed, Embase, and Cochrane Library databases were systematically searched for studies published until May 20, 2021. Included studies were prospective studies of patients with newly diagnosed PCNSL treated with ASCT. The pooled rates and 95% confidence intervals (CIs) were determined for all outcomes. Subgroup analysis was conducted to compare the relative risk (RR) with 95% CIs for the complete remission (CR) rate and the hazard ratios (HRs) with 95% CIs for progression-free survival (PFS) and overall survival (OS).

**Results:**

Thirteen prospective studies including 348 patients were analyzed. The pooled CR rate, overall response rate, and relapse rate were 80% (95% CI, 71–88%, *I^2^* = 67.06%, *p* = 0.00), 95% (95% CI, 87–100%, *I^2^* = 73.65%, *p*= 0.00), and 19% (95% CI, 15–24%, *I^2^* = 76.18%, *p* = 0.00), respectively. The pooled 2- and 5-year PFS and OS rates were 74% (95% CI, 68–80%, *I^2^* = 3.90%), 65% (95% CI, 51–77%, *I^2^* = 74.61%), 80% (95% CI, 72–88%, *I^2^* = 57.54%), and 69% (95% CI, 53–83%, *I^2^* = 83.89%), respectively. Hematological toxicity and infections were more common adverse events above grade 3. The pooled treatment-related mortality was 3% (95% CI, 1–6%, *I^2^* = 28.18%, *p* = 0.16). In the group analysis of ASCT compared with whole-brain radiotherapy, there were no significant differences in the CR rate (RR, 1.00, 95% CI, 0.88–1.14, *p* = 0.971), relapse rate (RR, 0.44, 95% CI, 0.06–3.10, *p* = 0.408), PFS (HR, 1.28, 95% CI, 0.81–2.01, *p* = 0.29), or OS (HR, 1.62, 95% CI, 0.97–2.69, *p* = 0.06). Cognitive functions were preserved or improved after ASCT.

**Conclusions:**

ASCT is a feasible approach for consolidation with good tolerability for newly diagnosed PCNSL patients. High-quality randomized controlled trials are still needed to confirm the effects of ASCT.

**Systematic Review Registration:**

https://www.crd.york.ac.uk/prospero/, identifier CRD42021268422.

## 1 Introduction

Primary central nervous system lymphoma (PCNSL) is a rare form of aggressive extranodal non-Hodgkin lymphoma located in the brain, leptomeninges, spinal cord, and intraocular structures (1, 2). The incidence of PCNSL has markedly increased in immunocompetent patients over the previous decades (3). Although high-dose methotrexate (HD-MTX)-based chemotherapy regimens significantly improved the prognosis of PCNSL, the duration of the remission period was short, and the disease easily progressed and recurred. The main therapeutic goals in the treatment of PCNSL are to further control the disease and prolong survival with consolidation therapy after induced remission.

Both whole-brain radiotherapy (WBRT) and high-dose chemotherapy (HDC) are important consolidation therapies in the management of PCNSL (4–9). Compared with WBRT alone, HD-MTX-based chemotherapy combined with WBRT extended the median progression-free survival (PFS) and overall survival (OS) by two to three times. However, WBRT can lead to irreversible neurotoxicity, such as brain dysfunction, progressive dementia, and urinary incontinence (10, 11). PCNSL mostly occurs in elderly individuals who have poor tolerance to high-dose combined chemotherapy (12, 13). Alternative strategies are being investigated to improve disease outcomes and mitigate neurocognitive side effects in patients. These include reduced-dose WBRT, nonmyeloablative high-dose chemotherapy, and HDC with autologous stem-cell transplantation (ASCT). There are no randomized studies that have compared all of these consolidation regimens head to head.

Different alternatives have been proposed, with ASCT being the most investigated strategy. A number of studies suggesting the high efficacy of HDC supported by ASCT, with acceptable tolerability, mostly using thiotepa-based conditioning regimens, have been reported (14, 15). However, the level of evidence in this field is still low, and the existing literature mostly comprises retrospective studies and small study samples. In the current study, we conducted a meta-analysis using the available data on the complete remission (CR) rate, overall response rate (ORR), relapse rate, grade 3–4 toxic effect rate, treatment-related mortality (TRM), PFS, and OS to assess the efficacy of ASCT as part of the first-line treatment for newly diagnosed PCNSL.

## 2 Methods

### 2.1 Search Strategy

This systematic review and meta-analysis was registered with the International Prospective Register of Systematic Reviews (PROSPERO, number CRD42021268422), and the study was carried out in accordance with the Preferred Reporting Items for Systematic Reviews and Meta-Analysis (PRISMA) statement (16, 17).

We carefully searched academic databases (PubMed, EMBASE, and the Cochrane Library) to identify relevant studies from the date the database was established until May 20, 2021. The search typically included two key terms: “primary central nervous system lymphoma” and “autologous stem-cell transplantation.” The search strategy for each database is shown in **Supplementary Table 1**. The search was not restricted by region, race, age, or payment method. In addition, we searched the reference lists of the identified articles, original studies, and previous meta-analyses to identify other potential studies. The databases were searched, and the results were imported to EndNote software (X9 version).

### 2.2 Inclusion and Exclusion Criteria

Studies were included according to the following criteria: (1) the study type included randomized clinical trials (RCTs), prospective cohort studies, and single-arm studies; (2) the patients were newly diagnosed with PCNSL confirmed by histopathology; (3) studies included PCNSL patients treated with ASCT as part of first-line treatment; (4) all the included studies reported sufficient data on survival outcomes, such as CR, PFS, and OS; and (5) for duplicate data or data from sequential publications, only the largest or newest research study was included.

Studies were excluded based on the following criteria: (1) written in a language other than English; (2) incomplete data for the targeted outcomes; (3) reviews, letters, reports, conference abstracts or papers, mail articles, editorials, and cellular or animal studies; (4) sample cases from a database; and (5) not related to the topic.

### 2.3 Data Extraction

Two investigators (JL and JG) independently extracted the data using a standardized form and subsequently validated the extracted data through discussion and consensus. For each study, the following data were collected: (1) last name of the first author, publication year, and study design; (2) study population location, sample size, median age, the sex of patients, induction chemotherapy, and median follow-up time; (3) toxicities associated with ASCT and TRM; and (4) survival outcomes, including CR, ORR (including the CR rate and partial remission rate), PFS, and OS. The data were directly collected from the article if the hazard ratios (HRs), 95% confidence intervals (CIs), and *p*-values were reported; otherwise, we extracted the data from Kaplan–Meier curves using Engauge Digitizer version 13.0 (18) or contacted the corresponding authors to obtain these data.

### 2.4 Quality Assessment

The quality of the included articles was assessed by two investigators (JL and JG) independently. We used the Cochrane Collaboration risk of bias tool to evaluate the included RCTs according to the Cochrane Handbook recommendations (19). The quality assessment of prospective cohort studies were evaluated by the modified Newcastle–Ottawa scale (20). Prospective nonrandomized studies were evaluated by the methodological index for nonrandomized studies (MINORS) (21).

### 2.5 Statistical Analysis

Statistical analysis was performed with STATA SE 15.1 (StataCorp, College Station, TX, USA) and RevMan 5.3 by two investigators (JL and JG) independently. Disagreements were resolved by discussion with the third investigator (XS). For the pooled rates, a random-effect model or a fixed-effect model with double arcsine transformation was used. The effect size (ES) of all combined results is represented by the 95% CI (with upper and lower limits).

Heterogeneity was assessed by the chi-square test (*Q*-statistic) and *I* ^2^ statistic. If *p* > 0.10 and/or *I* ^2^ < 50%, the heterogeneity was deemed to be low, and a fixed-effect (Mantel–Haenszel method) model was used; otherwise, we selected a random-effect (Mantel–Haenszel method) model because of the presence of significant heterogeneity. The ES for each meta-analysis was calculated as follows: (1) For all included studies, the CR rate, ORR, relapse rate, grade 3–4 toxic effect rate, and TRM are represented as pooled rates with corresponding 95% CIs. (2) For RCTs, the CR rate is represented as the relative risk (RR) with 95% CIs and the PFS and OS rates are represented as HRs with 95% CIs; if RR > 1.0, HR < 1.0, and *p* < 0.05, the results favored ASCT therapy and were considered statistically significant. In addition, we assessed publication bias by funnel plots, Begg’s test and Egger’s test.

## 3 Results

### 3.1 Study Selection and Characteristics

**Figure 1:**
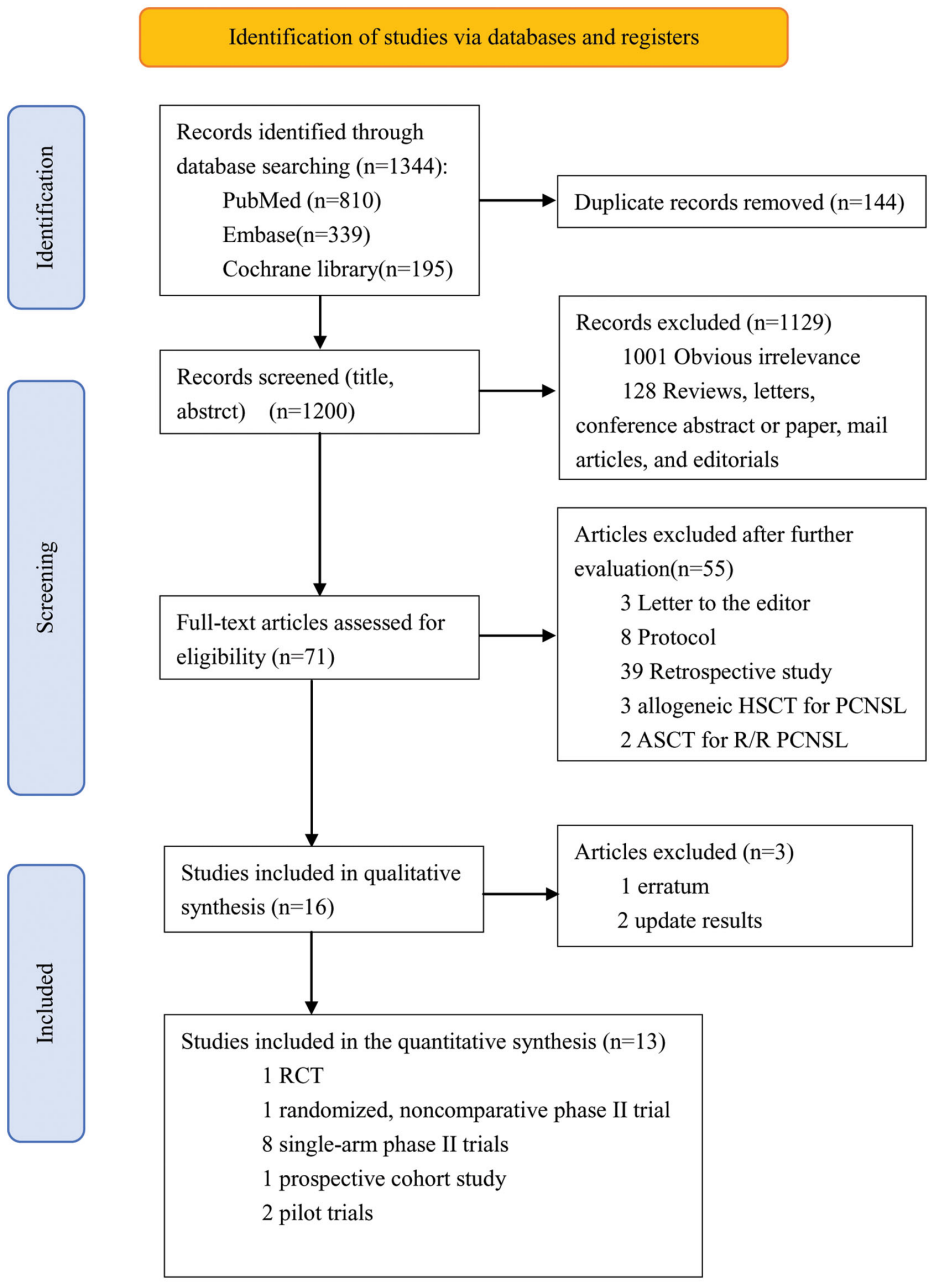
Flow diagram of the study selection process.

The flow chart of the screening process is shown in **Figure 1**. A total of 1,344 articles were screened by the search strategy, and 144 duplicate articles were excluded. Then, 1,200 articles underwent a title and abstract review, and 1,129 were excluded for the following reasons: obvious irrelevance (*n* = 1,001) and reviews, letters, conference abstracts or papers, mail articles, and editorials (*n* = 128). The remaining 71 articles were comprehensively reviewed, 55 of which were excluded for the following reasons: letter to the editor (*n* = 3), protocol (*n* = 8), retrospective study (*n* = 39), allogeneic HSCT for PCNSL (*n* = 3), and ASCT for relapse/refractory PCNSL (*n* = 2). The 16 remaining studies fulfilled the eligibility criteria for qualitative synthesis. After further evaluation, one study involved an error correction (22), and two studies updated the results of three studies (23, 24). Our meta-analysis ultimately included 13 articles, including 1 RCT (25), 1 randomized noncomparative phase II trial (26), 8 single-arm phase II trials (15, 27–33), 1 prospective cohort study (34), and 2 pilot trials (35, 36).

In the included studies, a total of 524 newly diagnosed PCNSL patients were included, 348 of whom received therapy with ASCT. The classification and features of the included studies, including the study design, publication year, recruitment period, country, sample size (total/number of patients who underwent ASCT), sex (male/female), median age, induction therapy, ASCT setting, and outcome indicators, are shown in **Table 1**. The 13 prospective studies were conducted in different countries: three were conducted in the United States, one was conducted in Europe, one was conducted in South Korea, two were conducted in France, five were conducted in Germany, and one was conducted in Canada. The MTX-based regimen was the most common induction therapy. The conditioning regimens included three studies with TBC (thiotepa, busulfan, cyclophosphamide), one study with rituximab-TBC, six studies with thiotepa combined with other drugs, one study with BUCYE (busulfan, cyclophosphamide, etoposide), and two studies with BEAM (carmustine, etoposide, cytarabine, melphalan) regimens, followed by ASCT.

**Table 1:** Characteristics of enrolled studies.

Reference	Year	Study Design	Recruitment Period	Country	Sample Size (ASCT)	Median Age, Years (Range)	Induction Therapy	ASCT Setting	Outcome Indicators
(25)	2017	RCT	2010–2014	Europe	ITT 118 (59)	58 (26–70)	MA or MAR or MART	T, Carmustine	CR, PFS, OS
(26)	2019	Randomized, noncomparative trial	2008–2014	France	ITT 132 (66)	55 (25–60)	R-MBVP + RA	TBC	CR, ORR, PFS, OS
(29)	2003	Single-arm phase II trial	NR	America	28 (14)	53 (25–71)	MA	BEAM	CR, ORR, OS
(31)	2015	Single-arm phase II trial	2005–2011	United States	32 (26)	57 (23-67)	R-MPV	TBC	CR, ORR, PFS, OS
(30)	2015	Single-arm phase II trial	NR	United States	30 (12)	54 (24-69)	MR ± A	R-TBC	CR, ORR
(28)	2016	Single-arm phase II trial	2007–2011	Germany	79 (73)	56 (51-62)	MR;	R, T, Carmustine	CR, ORR, PFS, OS
(32)	2006	Single-arm phase II trial	1999–2001	France	25 (17)	51 (21-60)	MBVP	BEAM	CR, ORR
(34)	2011	Prospective cohort study	2005–2008	South Korea	11 (11)	52 (33-65)	MA	BUCYE	CR, ORR, OS
(33)*	2007	Single-arm phase II trial	1999–2004	Germany	23 (16)	55 (18–69)	M	T, Busulfan,	CR, ORR, OS
(27)^#^	2006	Single-arm phase II trial	1998–2003	Germany	30 (23)	54 (27-64)	M	T, Carmustine	CR, ORR, OS
(15)^#^	2008	Pilot trial	2003–2006	Germany	13 (11)	54 (38-67)	M	TA	CR, ORR, OS
(35)	2003	Pilot trial	1998–2002	Canada	7 (7)	56 (41-64)	MA ± procarba- zine	TBC	CR, ORR
(36)	2020	Single-arm pilot trial	2015–2017	Germany	14 (13)	74 (69-79)	MR ± A	T, Busulfan	CR, ORR, PFS, OS

NR, not reported; ITT, intention-to-treat; M, methotrexate; A, cytarabine; R, rituximab; T, thiotepa; MBVP, M + etoposide + carmustine + prednisone/methylprednisolone; R-MPV, R + M + procarbazine + vincristine; TBC, T+ busulfan + cyclophosphamide; BEAM, carmustine + etoposide + A + melphalan; BUCYE, busulfan + cyclophosphamide + etoposide; *The results updated by Kiefer (23); ^#^The results were updated by Kasenda (24). CR, complete remission; ORR, overall response rate; OS, overall survival; PFS, progression-free survival.

### 3.2 Quality Assessment

The Cochrane Collaboration risk of bias tool was used to assess the quality of one included RCT (**Supplementary Figure 1**). The risk of bias graph showed that the included RCT was a high-quality study. Eight single-arm studies, one randomized noncomparative phase II trial, and two pilot trials were assessed using the MINORS index score, which ranges from 10 to 20 points and showed that these studies were acceptable for the present meta-analysis (**Table 2A**). In addition, we used the modified Newcastle–Ottawa scale for the quality assessment of one included prospective cohort study. This study was considered to be of high quality, with a rating of eight stars (**Table 2B**).

Table 2Quality assessment of included studies.

**A. MINORS index for included non-randomized studies.** 

Study	I	II	III	IV	V	VI	VII	VIII	IX	X	XI	XII	Total
Houillier et al. (26)	2	2	2	2	1	2	2	2	1	1	1	2	20
Abrey et al. (29)	2	2	2	2	0	2	2	0					12
Omuro et al. (31)	2	2	2	2	0	2	2	2					14
Chen et al. (30)	2	2	2	2	0	2	1	0					11
Illerhaus et al. (28)	2	2	2	2	0	2	2	1					13
Colombat et al. (32)	2	2	2	1	0	2	2	0					11
Montemurro et al. (33)	2	2	2	2	0	2	2	0					12
Illerhaus et al. (27)	2	2	2	2	0	2	2	1					13
Illerhaus et al. (15)	2	2	2	2	0	2	0	0					10
Schorb et al. (36)	2	2	2	2	0	2	2	2					14
Cheng et al. (35)	2	2	2	1	0	2	2	0					11

Numbers for I–VIII in heading refer to: I, a clearly stated aim; II, inclusion of consecutive patients; III, prospective collection of data; IV, endpoints appropriate to the aim of the study; V, unbiased assessment of the study endpoint; VI, follow-up period appropriate to the aim of the study; VII, loss to follow-up less than 5%; VIII, prospective calculation of the study size. Numbers for IX–XII in heading are additional criteria for comparative studies, which refer to: IX, an adequate control group; X, contemporary groups; XI, baseline equivalence of groups; XII, adequate statistical analyses.

**B. Newcastle-Ottawa Scale (NOS) for included cohort study.** 

	Selection			Comparability			Outcome	
Study	Q1	Q2	Q3	Q4	Q1	Q1	Q2	Q3
Yoon et al. (34)	☆		☆	☆	☆☆	☆	☆	☆

Numbers for Q1–Q4 in heading Selection refer to: Q1, representative of the ASCT group; Q2, representative of the non-ASCT group; Q3, ascertainment of treatment; Q4, outcome present at start of study. Number for Q1 in heading Comparability refers to: Q1, comparability of cohorts on the basis of the design or analysis. Matching: 1. Age, 2. HIV status, 3. ECOG status, 4. Involvement, 5. CSF protein, 6. Gender ratio, 7. Count, 8. Deep lesions. Numbers for Q1–Q3 in heading Outcome refer to: Q1, assessment of outcome; Q2, adequate follow-up; Q3, complete follow-up.

### 3.3 Efficacy

#### 3.3.1 Tumor Response

The 13 included studies reported the CR rate and relapse rate, and 12 studies reported the ORR.

**Figure 2:**
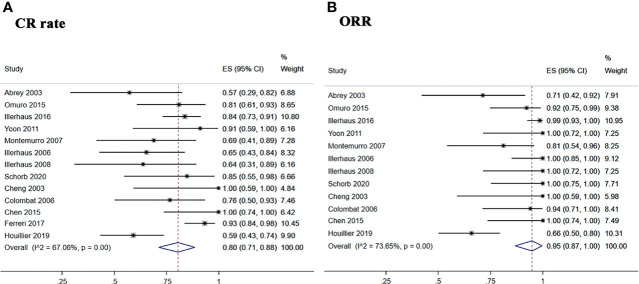
Forest plot of pooled response rate. **(A)** Complete remission rate. **(B)** overall response rate.

**Figure 3:**
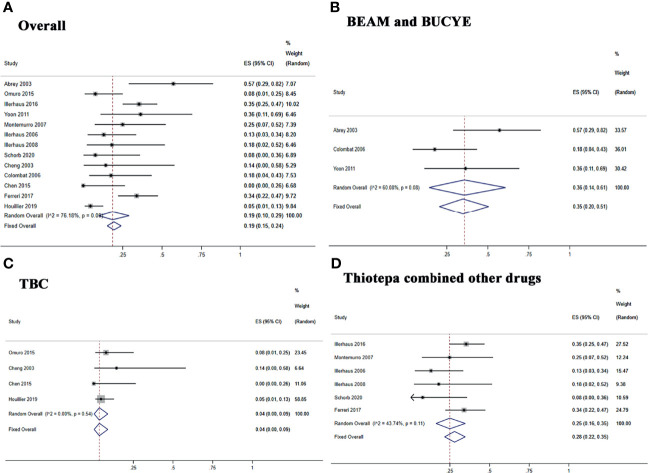
Forest plot of the relapse rate for treatment with the ASCT. **(A)** Overall relapse rete. **(B)** BEAM and BUCYE conditioning regimens. **(C)** TBC conditioning regimen. **(D)** Thiotepa combined other drugs.

The pooled CR rate, ORR, and relapse rate after treatment with ASCT were 80% (95% CI, 71–88%, *I^2^* = 67.06%, *p* = 0.00), 95% (95% CI, 87–100%, *I^2^* = 73.65%, *p* = 0.00), and 19% (95% CI, 15–24%, *I^2^* = 76.18%, *p* = 0.00), respectively (**Figure 2** and **Figure 3A**).

The subgroup analysis was stratified according to the different conditioning regimens. Three studies (29, 32, 34) investigated 42 PCNSL patients treated with the BEAM or BUCYE conditioning regimen, and the pooled relapse rate was 35% (95% CI, 20–51%, *I^2^* = 60.08%, *p* = 0.08). Four studies (26, 30, 31, 35) treated 111 patients with the TBC regimen, and the pooled relapse rate was 4% (95% CI, 0–9%, *I^2^* = 0%, *p* = 0.54). Six studies (15, 25, 27, 28, 33, 36) showed 195 patients with thiotepa combined with other drugs; the pooled relapse rate was 28% (95% CI, 22–35%, *I^2^* = 43.74%, *p* = 0.11) (**Figures 3B–D**).

#### 3.3.2 Survival

Five studies reported 1-, 2-, and 3-year PFS, and four studies reported 5-year PFS Kaplan–Meier curves. Ten studies reported 1-, 2-, and 3-year OS, and six studies reported 5-year OS Kaplan–Meier curves.

**Figure 4:**
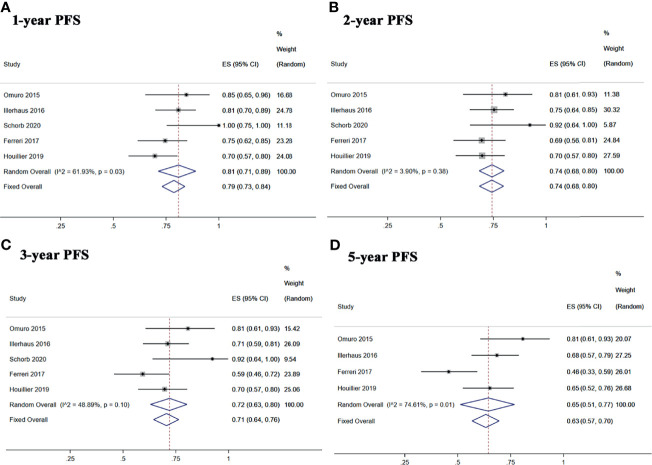
Forest plot of progression-free survival for treatment with ASCT. **(A)** 1-year progression-free survival. **(B)** 2-year progression-free survival. **(C)** 3-year progression-free survival. **(D)** 5-year progression-free survival.

**Figure 5:**
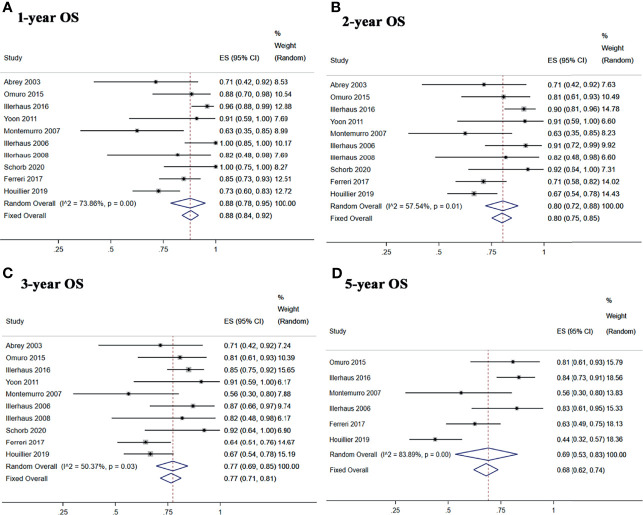
Forest plot of overall survival for treatment with ASCT. **(A)** 1-year overall survival. **(B)** 2-year overall survival. **(C)** 3-year overall survival. **(D)** 5-year overall survival.

The 1-, 2-, 3-, and 5-year pooled PFS rates for newly diagnosed PCNSL with ASCT were 79% (95% CI, 73–84%, *I* ^2^ = 61.93%, *p* = 0.03), 74% (95% CI, 68–80%, *I* ^2^ = 3.90%, *p* = 0.38), 71% (95% CI, 64–76%, *I* ^2^ = 48.89%, *p* = 0.10), and 65% (95% CI, 51–77%, *I* ^2^ = 74.61%, *p* = 0.01), respectively (**Figure 4**). The 1-, 2-, 3-, and 5-year pooled OS rates for newly diagnosed PCNSL with ASCT were 88% (95% CI, 78–95%, *I* ^2^ = 73.86%, *p* = 0.00), 80% (95% CI, 72–88%, *I* ^2^ = 57.54%, *p* = 0.01), 77% (95% CI, 69–85%, *I* ^2^ = 50.37%, *p* = 0.03), and 69% (95% CI, 53–83%, *I* ^2^ = 83.89%, *p* = 0.00), respectively (**Figure 5**).

**Figure 6:**
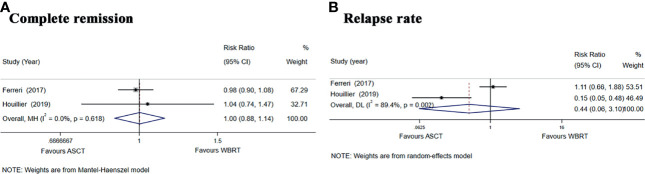
Forest plot of the CR and relapse rate for treatment with the ASCT group vs. WBRT group. **(A)** Complete remission rate. **(B)** Relapse rate.

We also performed a subgroup analysis of the two randomized phase II trials (25, 26) comparing the treatment with ASCT and WBRT for newly diagnosed PCNSL patients. We chose the Mantel–Haenszel fixed-effects model for the CR rate analysis because of the low heterogeneity among the included studies (*I* ^2^ = 0.0%, *p* = 0.618) and chose the Mantel–Haenszel random-effects model for the relapse rate analysis because of the high heterogeneity (*I* ^2^ = 89.4%, *p* = 0.002). The pooled data of the studies showed no significant difference in the CR rate (RR: 1.00, 95% CI, 0.88–1.14, *p* = 0.971) or the relapse rate (RR: 0.44, 95% CI, 0.06–3.10, *p* = 0.408) (**Figure 6**).

**Figure 7:**
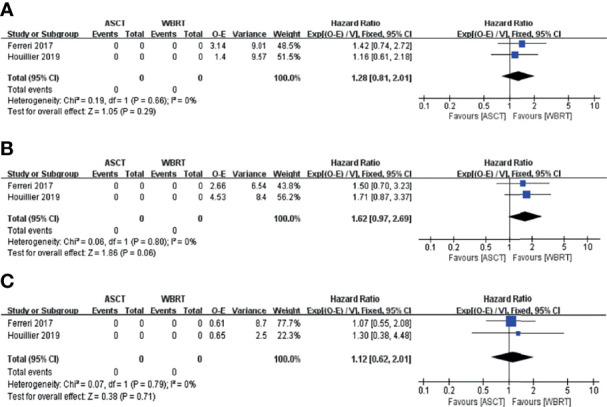
Forest plot of the survival for the treatment with the ASCT group vs. WBRT group. **(A)** Progression-free survival in the intention-to-treat analyses. **(B)** Overall survival in the intention-to-treat analyses. **(C)** Progression-free survival in the per-protocol analyses.

In the intention-to-treat analyses, the pooled analysis showed no significant differences in the PFS (HR = 1.28, 95% CI, 0.81–2.01, *p* = 0.29), with low heterogeneity (*I* ^2^ = 0%, *p* = 0.66) and OS (HR = 1.62, 95% CI, 0.97–2.69, *p* = 0.06), with low heterogeneity (*I* ^2^ = 0%, *p* = 0.80) (**Figures 7A, B**). For the 1-, 2-, and 5-year PFS, the RR for the ASCT group vs. WBRT group were 0.93 (95% CI, 0.80–1.07, *p* = 0.307), with low heterogeneity (*I* ^2^ = 0.0%, *p* = 0.462), 1.02 (95% CI, 0.74–1.40, *p* = 0.912), with high heterogeneity (*I* ^2^ = 72.5%, *p* = 0.057), and 1.34 (95% CI, 0.54–5.35, *p* = 0.531), with high heterogeneity (*I* ^2^ = 91.2%, *p* = 0.001) (**Supplementary Figure 2**). For 1- and 2-year OS, the pooled results favored the WBRT group (RR = 0.87, 95% CI, 0.78–0.97, *p* = 0.04), with low heterogeneity (*I* ^2^ = 32.1%, *p* = 0.225), and (RR = 0.86, 95% CI, 0.74–0.99, *p* = 0.038), with low heterogeneity (*I* ^2^ = 0.00%, *p* = 0.756). The pooled results showed no significant difference in the 5-year OS (RR = 0.86, 95% CI, 0.69–1.07, *p* = 0.156), with low heterogeneity (*I* ^2^ = 0.0%, *p* = 0.602) (**Supplementary Figure 3**).

In the per-protocol analyses, the pooled analysis showed no significant differences in the PFS (HR = 1.12, 95% CI, 0.62–2.01, *p* = 0.71), with low heterogeneity (*I* ^2^ = 0%, *p* = 0.79; **Figure 7C**). For the 1-, 2-, and 5-year PFS, the RR for the ASCT group vs. WBRT group were 0.99 (95% CI, 0.88–1.12, *p* = 0.878), with low heterogeneity (*I* ^2^ = 0.0%, *p* = 0.455), 1.14 (95% CI, 0.87–1.49, *p* = 0.356), with high heterogeneity (*I* ^2^ = 67.7%, *p* = 0.078), and 1.23 (95% CI, 0.65–2.34, *p* = 0.523), with high heterogeneity (*I* ^2^ = 85.8%, *p* = 0.008), respectively (**Supplementary Figure 4**).

#### 3.3.3 Toxicity

**Figure 8:**
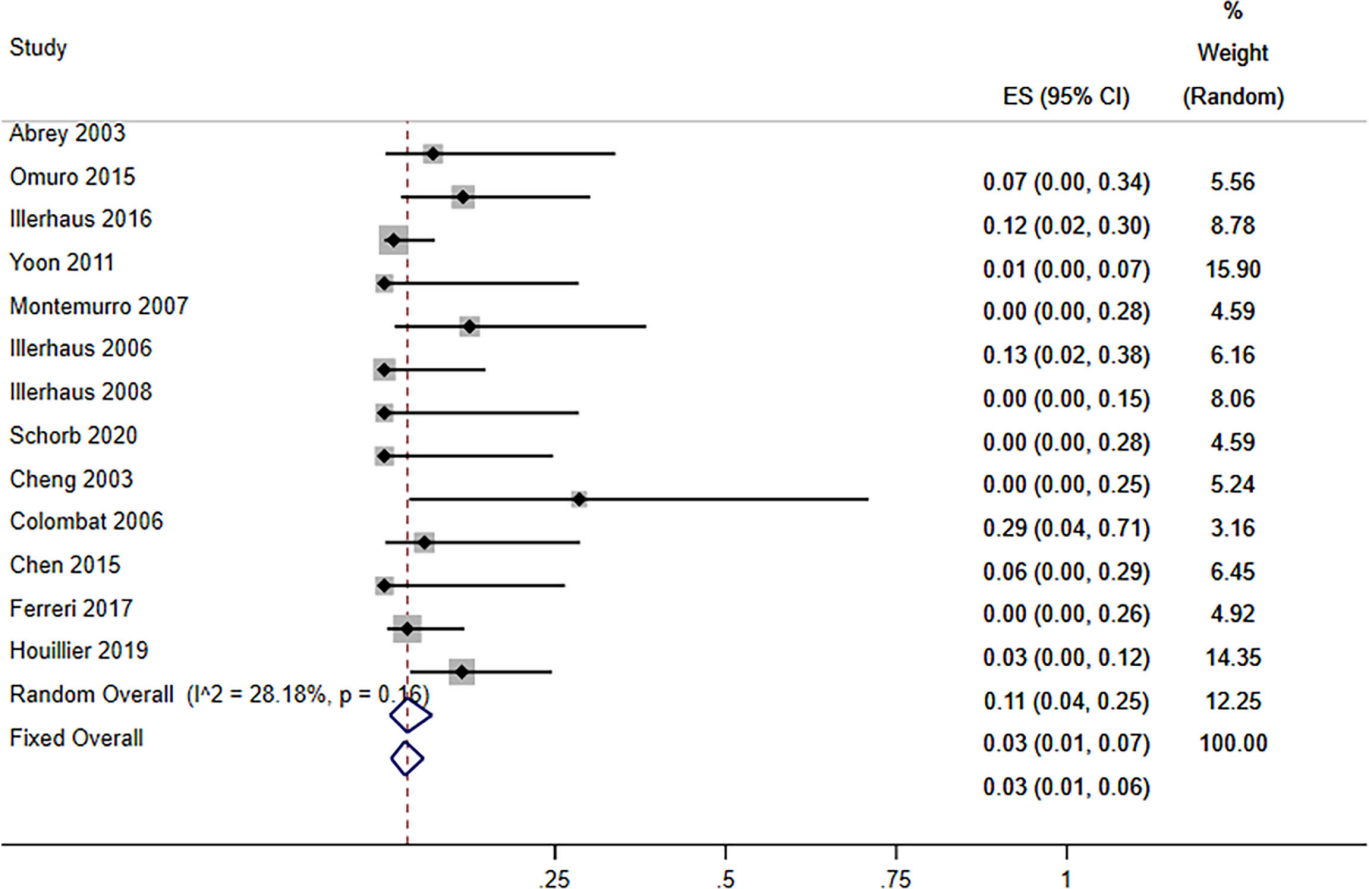
Forest plot of the treatment-related mortolity for treatment with the ASCT.

For the grade 3–4 toxic effect rate, we performed a subgroup analysis of the included studies based on the type of side effect. Hematologic toxicities mainly included neutropenia/leukopenia, thrombocytopenia, and anemia. The pooled rate of grade 3–4 hematologic toxicities was 99% (95% CI, 97–100%, *I* ^2^ = 0.0%, *p* = 0.68). Severe nonhematologic toxicities mainly included infection, febrile neutropenia, hepatotoxicity, gastrointestinal toxicities, mucositis, and acute neurotoxicity/encephalopathy. The pooled rate of grade 3–4 febrile neutropenia or infection was 59% (95% CI, 53–65%, *I* ^2^ = 94.15%, *p* = 0.00). The pooled rate of grade 3–4 hepatotoxicity was 4% (95% CI, 1–8%, *I* ^2^ = 0.0%, *p* = 0.75). The pooled rate of grade 3–4 gastrointestinal toxicities was 16% (95% CI, 11–22%, *I* ^2^ = 0.0%, *p* = 0.62). The pooled rates of grade 3–4 mucositis and acute neurotoxicity/encephalopathy were 28% (95% CI, 22–34%, *I* ^2^ = 92.55%, *p* = 0.00) and 4% (95% CI, 1–8%, *I* ^2^ = 67.09%, *p* = 0.00), respectively (**Table 3**). The pooled TRM was 3% (95% CI, 1–6%, *I* ^2^ = 28.18%, *p* = 0.16) (**Figure 8**).

**Table 3:** Pooled results of common AEs of ≥grade 3.

Adverse Event	≥Grade 3	
	Effect Size (%95 CI)	*I* ^2^ (%), *P*
Hematologic toxicities	0.99 (0.97, 1.00)	0.00, 0.68
Febrile neutropenia or infections	0.59 (0.53, 0.65)	94.15, 0.00
Hepatotoxicity	0.04 (0.01, 0.08)	0.00, 0.75
Gastrointestinal	0.16 (0.11, 0.22)	0.00, 0.62
Mucositis	0.28 (0.22, 0.34)	92.55, 0.00
Acute neurotoxicity/encephalopathy	0.04 (0.01, 0.08)	67.09, 0.00

### 3.4 Sensitivity Analysis

We conducted sensitivity analysis by removing individual studies one by one from the pooled results with high heterogeneity to check the influence of the removed data set on the overall ES. The results of the sensitivity analysis indicated that the pooled result was stable in terms of the CR rate, ORR, toxic effect, PFS, and OS analyses when studies were omitted, indicating that our combined results are reliable (**Supplementary Figures 5–9**).

### 3.5 Publication Bias

**Figure 9:**
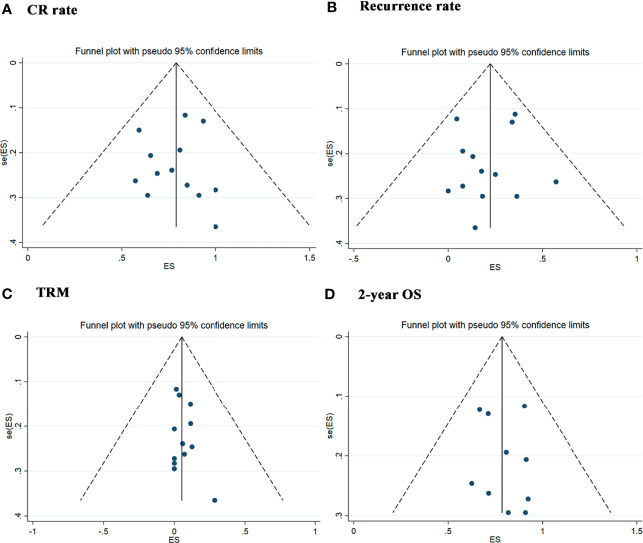
Publication Bias with the funnel plot. **(A)** Assessment result of the complete remission rate. **(B)** Assessment result of the recurrence rate. **(C)** Assessment result of the treatment-related mortolity. **(D)** Assessment result of the 2-year overall survival.

We used the funnel plot and Egger’s and Begg’s tests to evaluate the publication bias in studies assessing the CR rate (*p* = 0.714 for Begg’s test and *p* = 0.776 for Egger’s test), recurrence rate (*p* = 1.000 for Begg’s test and *p* = 0.643 for Egger’s test), TRM (*p* = 0.714 for Begg’s test and *p* = 0.438 for Egger’s test) and 2-year OS rate (*p* = 0.788 for Begg’s test and *p* = 0.669 for Egger’s test), which did not demonstrate significant publication bias (**Figure 9**).

## 4 Discussion

PCNSL has a dismal prognosis. Although there are therapeutic progress and improvement in survival figures (37), the outcomes of patients with PCNSL remain poor, with 20–30% survival at 5 years and 10–20% survival at 10 years (38–40). Induction treatment with HD-MTX-based chemotherapy is standard for PCNSL and can achieve a CR rate of more than 40% and an ORR of 60% or higher (12). Despite high response rates with initial HD-MTX-based treatment, approximately 50% responders are still at risk for progression or relapse. Moreover, approximately 25% patients fail to respond to the initial treatment (41). In a retrospective study, the median OS from the time of progression was 3.7 months for patients who relapsed within the first year of the initial therapy and 2 months for primary refractory patients (42).

Induction chemotherapy followed by consolidation therapy has been proposed. Consolidation treatment includes WBRT, HDC supported by ASCT, and nonmyeloablative chemotherapy. WBRT in the management of PCNSL has the risk of severe neurotoxicity observed in patients treated with chemoradiotherapy (43, 44). HDC supported by ASCT is the most commonly investigated alternative to WBRT in patients with PCNSL (45). Data are currently limited to retrospective studies and several single-arm phase II trials with few subjects enrolled, and the effects of ASCT on PCNSL are controversial.

In the current study, we conducted a meta-analysis to evaluate the efficacy and safety of HDC followed by ASCT as first-line treatment in newly diagnosed PCNSL patients. In all included studies, induction chemotherapy was based on HD-MTX either as monotherapy or combination, using drugs including cytarabine, carmustine, etoposide, procarbazine, rituximab, temozolomide, pemetrexed, thiotepa, and so on. Intensive chemotherapy mainly contained a thiotepa-based regimen. Our analysis of the efficacy of ASCT in the treatment of newly diagnosed PCNSL patients showed that the pooled CR rate and ORR could reach 80 and 95%, respectively. Only one included study (36) conducted the ASCT in elderly patients with a median age of 74 years (range, 69–79 years). This study showed an increase in CR rate from 28.6 to 84.6% pre- and post-ASCT. This finding shows that ASCT could achieve satisfactory results in the treatment of PCNSL. The relapse rate was 19% after ASCT treatment. HD-MTX-based chemotherapy followed by ASCT as consolidate treatment with higher response rate and lower relapse rate compared with induction chemotherapy alone.

However, the *I* ^2^ values for the overall CR rate, ORR, and relapse rate were 67.06, 73.65, and 76.18%, which was quite heterogeneous. The heterogeneity of response rate may be related to the difference of including induction chemotherapy and/or conditioning regimens: MTX-based combination chemotherapy and high-dose MTX (≥3.5 g/m^2^) with higher response rate than MTX monotherapy and MTX dose below 3.5 g/m^2^. The subgroup analysis stratified according to the different conditioning regimens significantly reduced the heterogeneity of relapse rate, indicating that the heterogeneity was derived from the differences in the conditioning treatment plan. The subgroup analysis showed that the relapse rate from BEAM/BUCYE, TBC, and thiotepa combined with other drugs (busulfan/carmustine/cytarabine) were 35, 4, and 28% respectively. The BEAM regimen is a feasible approach with good tolerability, especially for elderly patients with PCNSL. However, these drugs have poor blood–brain barrier permeability with higher relapse rate. Thiotepa, busulfan, and cyclophosphamide can penetrate the blood–brain barrier (46–48). Compared to the BEAM regimen, theoretically, TBC drugs should produce better effects because of good blood–brain barrier penetration. On one study in 2003 by Abrey et al. (29), 14 patients received the BEAM regimen, followed by ASCT. Eight patients (57.1%) experienced relapse a median of 2.3 months after transplantation (range, 1.3–29.6 months), all but one patient within 7 months. In 2011, Yoon et al. (34) reported that 11 patients received BUCYE as a conditioning regimen; four (36.3%) patients experienced relapse within 1 year. Although effective for systemic lymphoma, these findings have questioned the efficacy of BEAM as consolidation for PCNSL. The relapse rate ranged from 0 to 14.3% with TBC regimen (26, 30, 31, 35), which is much lower than that of the thiotepa combined with busulfan/carmustine/cytarabine (7.7–35%) (15, 25, 27, 28, 33, 36) and BEAM/BUCYE regimens (17.6–57.1%) (29, 32, 34). The best results in the Chen et al. study (30) are the TBC combined with rituximab as a conditioning regimen for 12 newly diagnosed PCNSL patients, none of whom experienced relapse or death.

The subgroup analysis of the two included randomized phase II studies, the pooled analysis of CR rate and relapse rate in the ASCT group compared with the WBRT group, suggested that there were no significant differences. In the International Extranodal Lymphoma Study Group-32 (IELSG32) study (25), 20 patients relapsed after ASCT, while only 3 relapsed in the PRECIS trial (26). The chemotherapy regimen was more intensive in the PRECIS trial, which might explain the apparently lower number of relapses after ASCT in the PRECIS study. The number of relapsed patients in the WBRT group (36 vs. 40 Gy) in the two trials was similar: 18 and 20.

After HD-MTX-based induction therapy, only 20–30% of patients achieve durable long-term remission (49, 50). In our study, the pooled survival analysis of ASCT treatment showed promising results. The pooled 1-, 2-, 3-, and 5-year PFS rates were 84, 80, 77, and 69%. The pooled 1-, 2-, 3-, and 5-year OS rates were 90, 85, 83, and 78%. The heterogeneity of 1- and 5-year PFS and 1-, 2-, 3-, and 5-year OS was relatively significant, which may be due to the limited sample size, various induction or intensive chemotherapy regimens used, and the different characteristics of the patients [age, Eastern Cooperative Oncology Group (ECOG) status, lesion depth, and so on]. The subgroup analysis of ASCT group compared with the WBRT group, the intention-to-treat analyses of the pooled 1- and 2-year OS rates showed higher OS rates in the WBRT group than in the ASCT group. Early ASCT treatment-related mortality because of hematological toxicities might explain the shorter OS than the WBRT group in the first and second years. In the per-protocol analyses, the pooled results of 2- and 5-year PFS tended to favor the ASCT group but with no significant differences.

We also conducted a meta-analysis of grade 3–4 toxicities and found that the most common toxicities were hematological toxicities and febrile neutropenia or infections. Hematological toxicities mainly included neutropenia or leukopenia, thrombocytopenia, and anemia, with an incidence of 99%. The nonhematological toxicities with the highest incidences were infection or febrile neutropenia (59%) and mucositis (28%). Some studies reported that WBRT had a high risk of neurotoxicity (6, 51). In this study, acute neurotoxicity/encephalopathy after ASCT treatment occurred in only 4% of patients. The TRM of ASCT was 3%. Nevertheless, the results of our study still suggest that clinicians should pay attention to the prevention of toxicities such as bone marrow suppression, infection, mucositis, hepatotoxicity, and gastrointestinal tract dysfunction when treating patients with ASCT.

Regarding the cognitive function of PCNSL patients after treatment, the result was consistent in the included studies (25, 26, 28, 29, 31, 35), suggesting that the cognitive function improved over time post-transplantation. Cognitive and quality-of-life (QoL) measures were proposed to be used in prospective clinical trials. Neuropsychological tests usually covered attention, executive functions, memory, and psychomotor speed (10). The Phase II trial in 2015 by Omuro et al. (31) evaluated neurotoxicity by neuropsychological testing, mood/QoL scores, and white matter changes on MRI over time (at baseline, after induction chemotherapy, and before transplant, every 6 months after transplant). Neuropsychological testing included TMTA (Trail-Making Test Part A), TMTB (TMT Part B), BTA (Brief Test of Attention), COWA (Controlled Word Association Test), HVLT-RTL (Hopkins Verbal Learning Test–Revised–Total Learning), HVLT-R-DEL (HVLT–Revised–Delayed Recall), HVLT-R-DI (HVLT–Revised–Discrimination Index), GPT-D (Grooved Pegboard Test–Dominant Hand), and GPT-ND (GPT–Non-Dominant Hand). Results of the HVLT-R-DEL and HVLT-R-DI tests indicated continuous improvement in scores from baseline over time. All of the other tests suggested that the rate of cognitive improvement slowed by 12–18 months post-transplant. Mood/QoL scores used BDI (Beck Depression Inventory) and FACT-BR (Functional Assessment of Cancer Therapy–Brain Cancer). BDI scores significantly and linearly decreased over time. FACT-BR scores significantly improved from baseline, with slowed improvement by 12–18 months post-transplant. An analysis of white matter abnormalities (modified Fazekas scale) showed an improvement (62%) after induction chemotherapy compared with baseline. Following transplant, there was an increase (25%) in white matter abnormalities compared with induction chemotherapy, and then they remained stable over time. In 2016, Illerhaus et al. (28) reported that the mean MMSE (Mini–Mental State Examination) score and the summary measure of Global Health Status Score (European Organisation for Research and Treatment of Cancer QoL core questionnaire) increased over time. Abrey et al. (29) showed that prospective neuropsychologic evaluations for patients with continued follow-up after ASCT displayed no evidence of significant delayed treatment-related neurocognitive decline. Cheng et al. (35) showed that patients experienced an improvement in their neurological performance and QoL post-transplantation. The two included randomized clinical trials demonstrated that cognitive impairment was observed after WBRT, whereas cognitive functions were preserved or improved after ASCT (25, 26). In the PRECIS trial (26), patients were evaluated on global cognitive function [MMSE and Mattis Dementia Rating Scale (MDRS)], episodic verbal memory [free and cued selective reminding test (FCSRT)], attention and mental flexibility (executive function; TMTA and TMTB), and psychoaffective status (motivation; Marin’s apathy scale) at baseline, after induction, and every 6 months after consolidation treatment, respectively. Neuropsychological testing showed an improvement in cognitive function at the end of induction chemotherapy in both groups. However, over time, patients presented a poorer score after WBRT and an improved score after ASCT in executive functions (MDRS, FCSRT total free recall, and TMT), whereas no significant change in hippocampus functions (FCSRT total free and cued recall) was observed in either group. In the IELSG32 trial (25), patients receiving consolidation treatment were assessed on cognitive functions and QoL. An analysis of delta values between baseline and post-treatment scores presented a significant improvement in attention and executive functions (TMT, phonemic verbal fluency) and visuoconstructive abilities (Rey Complex Figure Copy Test) in ASCT treatment. Moreover, the differences in scores between post-treatment immediately and at 2 years of follow-up after treatment showed a significant impairment in some attention and executive functions [Wisconsin card sorting test (WCST)] in the WBRT group, a remarkable improvement in these attention and executive functions, memory (Rey auditory verbal learning test–delayed recall), and QoL figures in the ASCT group. However, one study showed that the use of reduced-dose WBRT (23.4 Gy) resulted in stable cognitive functions for up to 2 years (52). As consolidation therapy, radiation doses are different in different studies, from 23 to 45 Gy (25, 26, 43, 52, 53), suggesting that the radiation dose is proportionately associated with the risk of neurotoxicity, whereas the relation between radiation dose reduction and efficacy remains to be defined.

Morris et al. (52) treated 52 newly diagnosed PCNSL patients with rituximab, HD-MTX, procarbazine, and vincristine as induction chemotherapy who achieved a CR received reduced-dose WBRT (23.4 Gy) and Ara-C. This trial showed high response rates, long-term disease control, and minimal neurotoxicity. In another trial [Radiation Therapy Oncology Group (RTOG) 0227] conducted by Glass et al. (54), patients received HD-MTX, rituximab, and temozolomide, followed by reduced-dose WBRT (36 Gy) and temozolomide with a favorable 2-year OS and PFS as well as the low incidence of late neurotoxicity, cognitive decline, decreased QoL, and leukoencephalopathy compared with the RTOG-9310 trial (WBRT: 45 Gy) (55). These results suggested that reduced-dose WBRT is feasible and effective as consolidation therapy after HD-MTX-based chemotherapy. However, the optimal dose remains to be defined and studies identifying the patients at greatest risk for neurotoxicity are clearly needed.

This meta-analysis has several limitations (1): There were only two randomized clinical trials (one RCT and one randomized noncomparative phase II trial) included because of the rarity of PCNSL. Single-arm trials make it difficult to compare ASCT treatment with other consolidation treatments. (2) All prospective studies were phase II clinical trials. (3) Some data extracted from the Kaplan–Meier curve may be biased. (4) The induction therapy or intensive chemotherapy regimens used among different institutions were significantly different. (5) Due to language limitations, this study only included studies in English.

## 5 Conclusions

At present, it is supported that PCNSL treatment includes induction chemotherapy, followed by one consolidation therapy. The HD-MTX-based regimen is the standard induction chemotherapy; however, it is not clear which consolidation therapy should be selected and which is the optimal choice. ASCT as a consolidation strategy is the most investigated approach. Through a meta-analysis of existing prospective studies on ASCT in newly diagnosed PCNSL patients, we found that ASCT can be safe and effective. The results are based on up-to-date information and a high quality of evidence according to the objective of this analysis. Nevertheless, in terms of the limitations of our study, it is essential to update and confirm the analysis in the future when there are more well-controlled, large-sample randomized clinical trials. Further studies are needed to determine the optimal intensive chemotherapy regimen, followed by ASCT and the optimal strategy for relapse after ASCT in the treatment of PCNSL.

## Data Availability Statement

The original contributions presented in the study are included in the article/**Supplementary Material**. Further inquiries can be directed to the corresponding authors.

## Author Contributions

YL and CG designed the study. JL, JG, and XS conducted literature search and data extraction and performed the statistical analysis. JL wrote the manuscript. CG assisted in writing the manuscript. YL and CG reviewed and edited the manuscript in detail. All authors contributed to the article and approved the submitted version.

## Funding

This work was supported by Capital’s Funds for Health Improvement and Research, NO.2020-2-2049.

## Conflict of Interest

The authors declare that the research was conducted in the absence of any commercial or financial relationships that could be construed as a potential conflict of interest.

## Publisher’s Note

All claims expressed in this article are solely those of the authors and do not necessarily represent those of their affiliated organizations, or those of the publisher, the editors and the reviewers. Any product that may be evaluated in this article, or claim that may be made by its manufacturer, is not guaranteed or endorsed by the publisher.
