# Supplementary material for: Efficacy and Safety of Autologous Stem-Cell Transplantation as Part of First-Line Treatment for Newly Diagnosed Primary Central Nervous System Lymphoma: A Systematic Review and Meta-Analysis
Source: Front Oncol. 2022 Jan 12;11:799721. doi: 10.3389/fonc.2021.799721 (PMC8790123; doi:10.3389/fonc.2021.799721)
Supplement: Supplementary file 1 [file DataSheet_1.docx]

Supplementary Material

# Supplementary Table

**Supplementary Table 1** The search strategy for each database

| Database | Search strategy |
| --- | --- |
| PubMed | #1. "central nervous system neoplasms"[MeSH Terms] |
|  | #2. "central nervous system"[Title/Abstract] OR "CNS"[Title/Abstract] |
|  | #3. "neoplasm*"[Title/Abstract] OR "lymphom*"[Title/Abstract] OR "tumor*"[Title/Abstract] OR "tumour*"[Title/Abstract] |
|  | #4. #2 AND #3 |
|  | #5. "primary central nervous system lymphoma"[Title/Abstract] OR (("primaries"[All Fields] OR "primary"[All Fields]) AND "central lymphoma"[Title/Abstract]) OR "PCNSL"[Title/Abstract] OR "primary cns lymphoma"[Title/Abstract] |
|  | #6. #4 OR #5 |
|  | #7. #1 OR #6 |
|  | #8. "hematopoietic stem cell transplantation"[MeSH Terms] |
|  | #9. "stem cell transplantation hematopoietic"[Title/Abstract] OR "transplantation hematopoietic stem cell"[Title/Abstract] OR "SCT"[Title/Abstract] OR "HSCT"[Title/Abstract] OR "ASCT"[Title/Abstract] OR "autologous stem cell transplantation"[Title/Abstract] OR "autologous stem cell transplant"[Title/Abstract] |
|  | #10. #8 OR #9 |
|  | #11. #7 AND #10 |
| Cochrane library | #1. MeSH descriptor: [Central Nervous System Neoplasms] explode all trees |
|  | #2. ("Central Nervous System"):ti,ab,kw OR (CNS):ti,ab,kw |
|  | #3. (neoplasm*):ti,ab,kw OR (tumor*):ti,ab,kw OR (tumour*):ti,ab,kw OR (lymphom*):ti,ab,kw |
|  | #4. #2 AND #3 |
|  | #5. ("primary central nervous system lymphoma"):ti,ab,kw OR ("primary central lymphoma"):ti,ab,kw OR ("primary CNS lymphoma"):ti,ab,kw OR (PCNSL):ti,ab,kw |
|  | #6. #1 OR #4 OR #5 |
|  | #7. (Stem Cell Transplantation, Hematopoietic):ti,ab,kw OR (Transplantation, Hematopoietic Stem Cell):ti,ab,kw OR (autologous stem-cell transplantation):ti,ab,kw OR (autologous stem cell transplant*):ti,ab,kw |
|  | #8. MeSH descriptor: [Hematopoietic Stem Cell Transplantation] explode all trees |
|  | #9. (SCT):ti,ab,kw OR (HSCT):ti,ab,kw OR (ASCT):ti,ab,kw |
|  | #10. #7 OR #8 OR #9 |
|  | #11. #6 AND #10 |
| Embase | #1. 'primary central nervous system lymphoma'/exp |
|  | #2. 'primary cns lymphoma':ab,ti OR pcnsl:ab,ti OR 'primary central lymphoma':ab,ti |
|  | #3. #1 OR#2 |
|  | #4. 'hematopoietic stem cell transplantation'/exp |
|  | #5. 'autologous hematopoietic stem cell transplantation':ab,ti OR sct:ab,ti OR asct:ab,ti OR hsct:ab,ti |
|  | #6. #4 OR #5 |
|  | #7. #3 AND #6 |

# Supplementary Figures

**
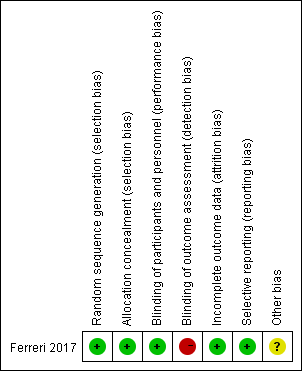
**

**Supplementary Figure 1.** Quality assesssment with the Cochrane Collaboration risk of bias tool for included RCT.


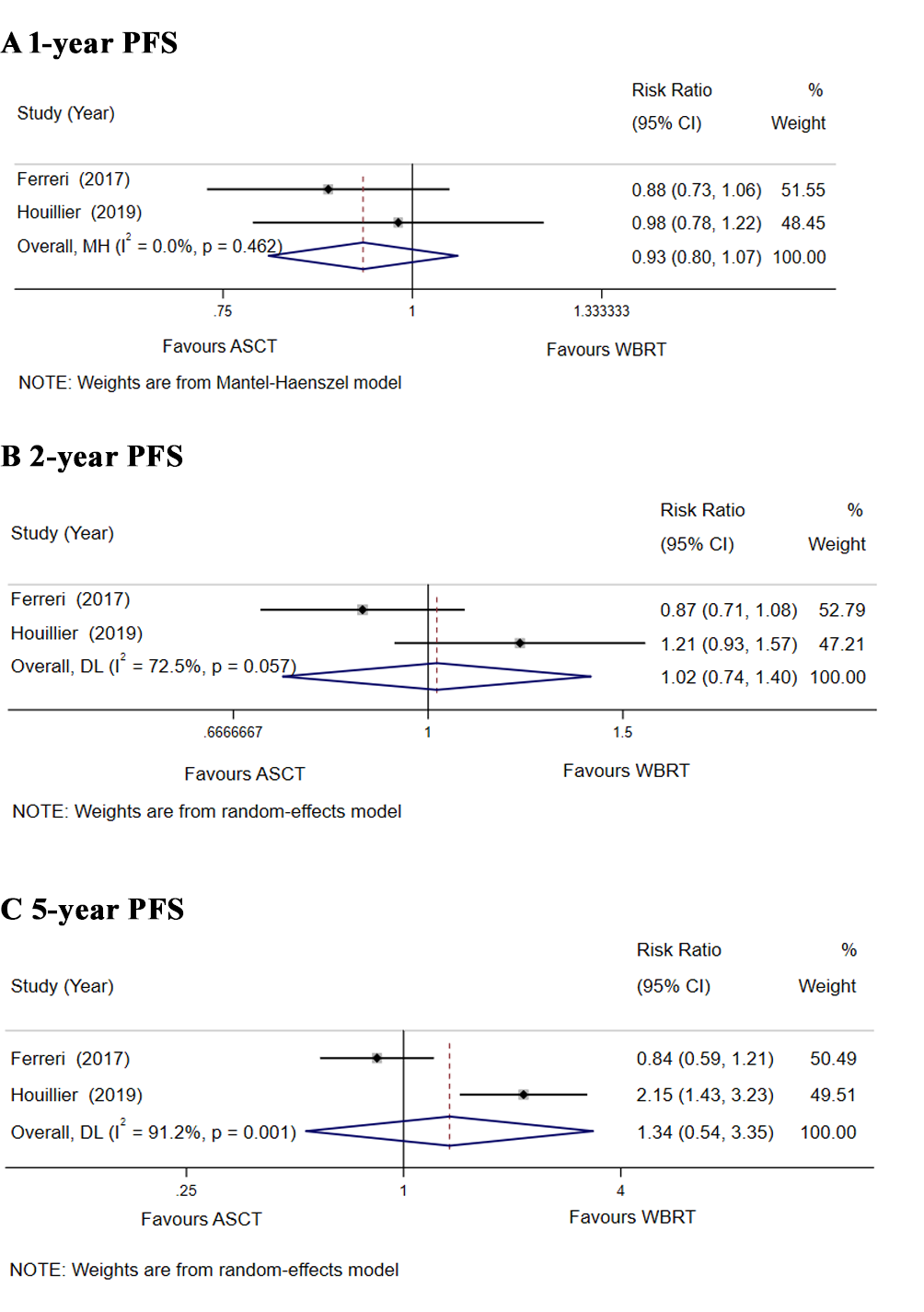


**Supplementary Figure 2.** Forest plot of the progression-free survival for treatment with the ASCT group vs. WBRT group in the intention-to-treat analyses. (A) 1-year progression-free survival. (B) 2-year progression-free survival. (C) 5-year progression-free survival.


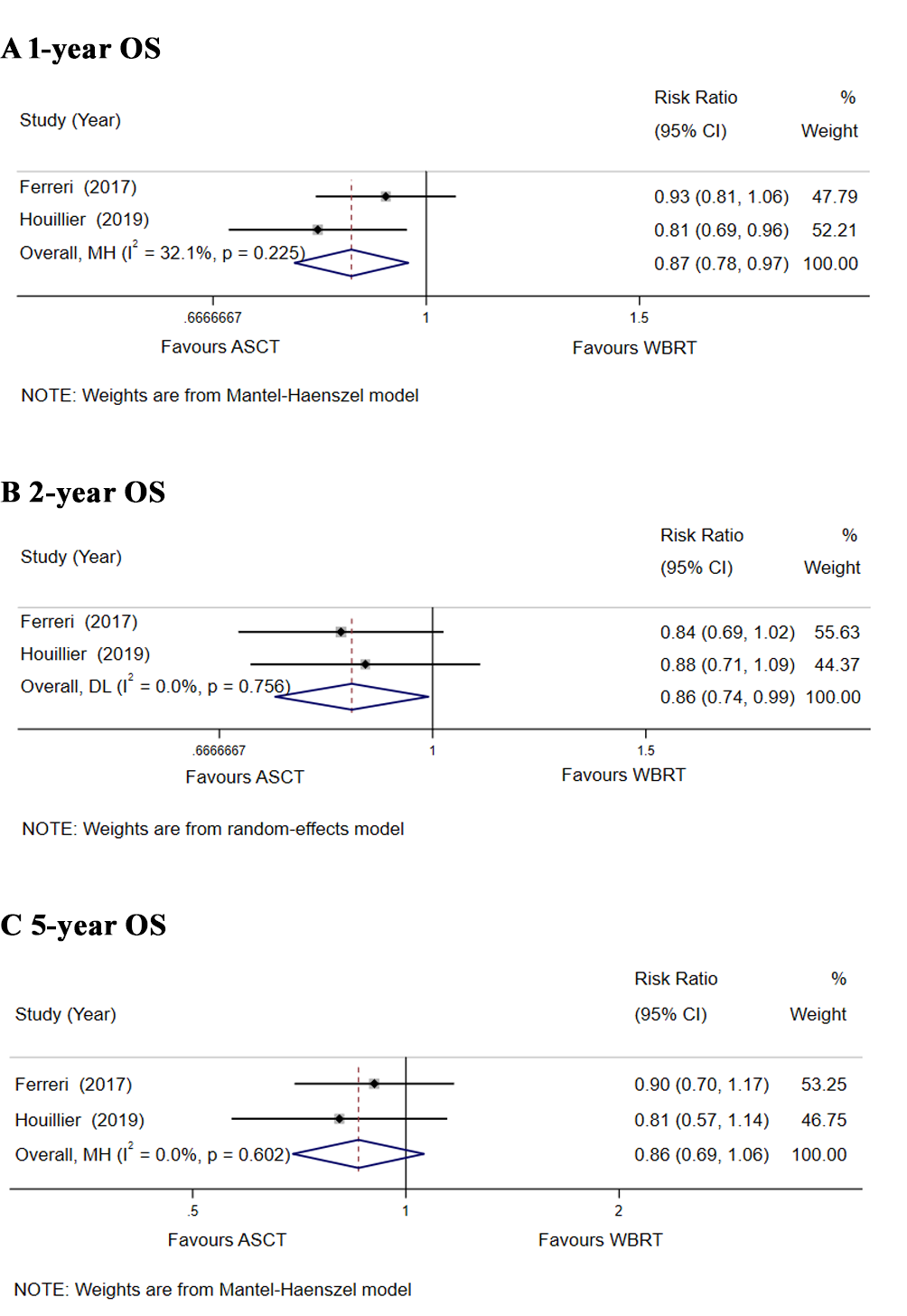


**Supplementary Figure 3.** Forest plot of the overall survival for treatment with the ASCT group vs. WBRT group in the intention-to-treat analyses. (A) 1-year overall survival. (B) 2-year overall survival. (C) 5-year overall survival.


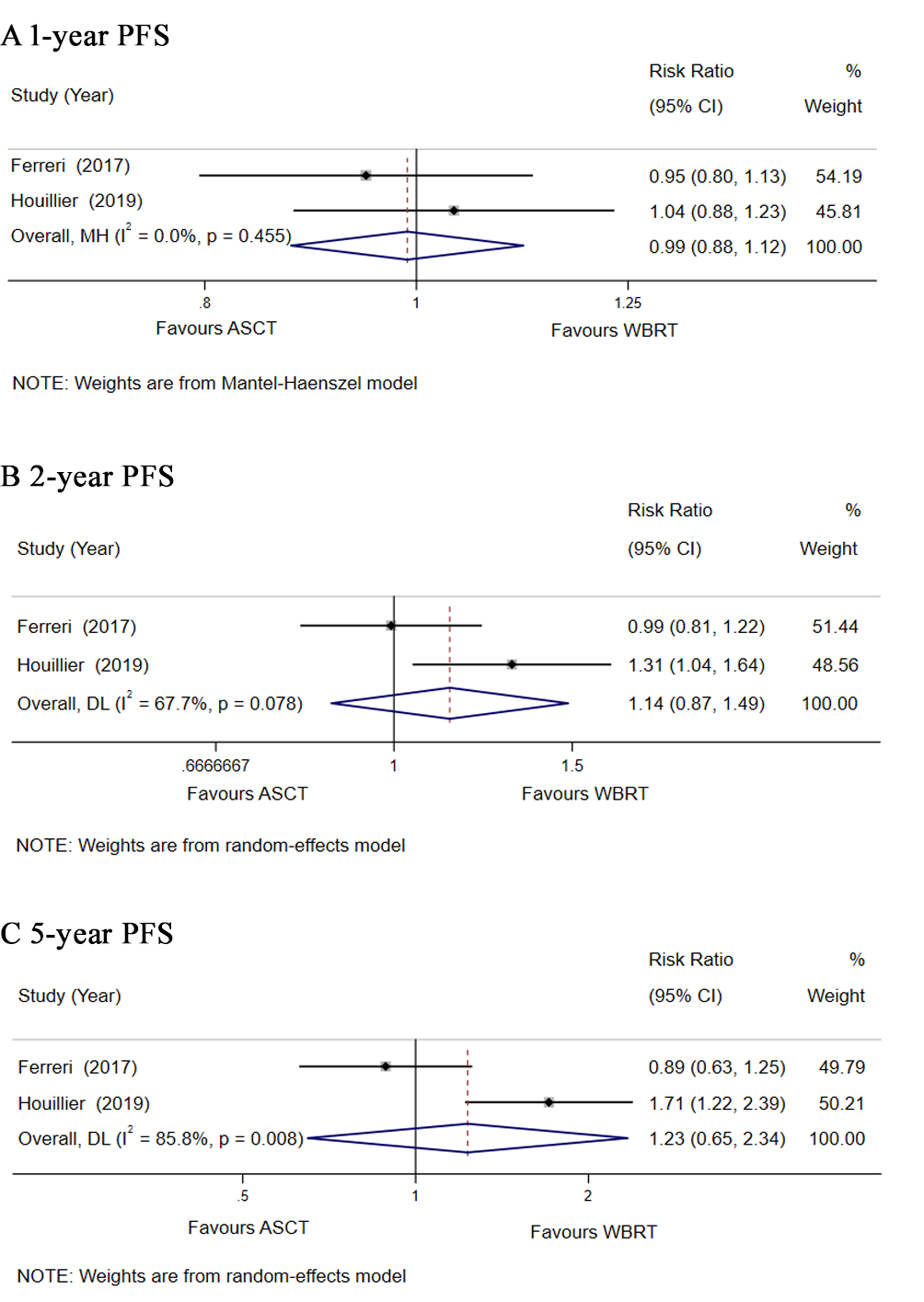


**Supplementary Figure 4.** Forest plot of the progression-free survival for treatment with the ASCT group vs. WBRT group in the per-protocol analyses. (A) 1-year progression-free survival. (B) 2-year progression-free survival. (C) 5-year progression-free survival.


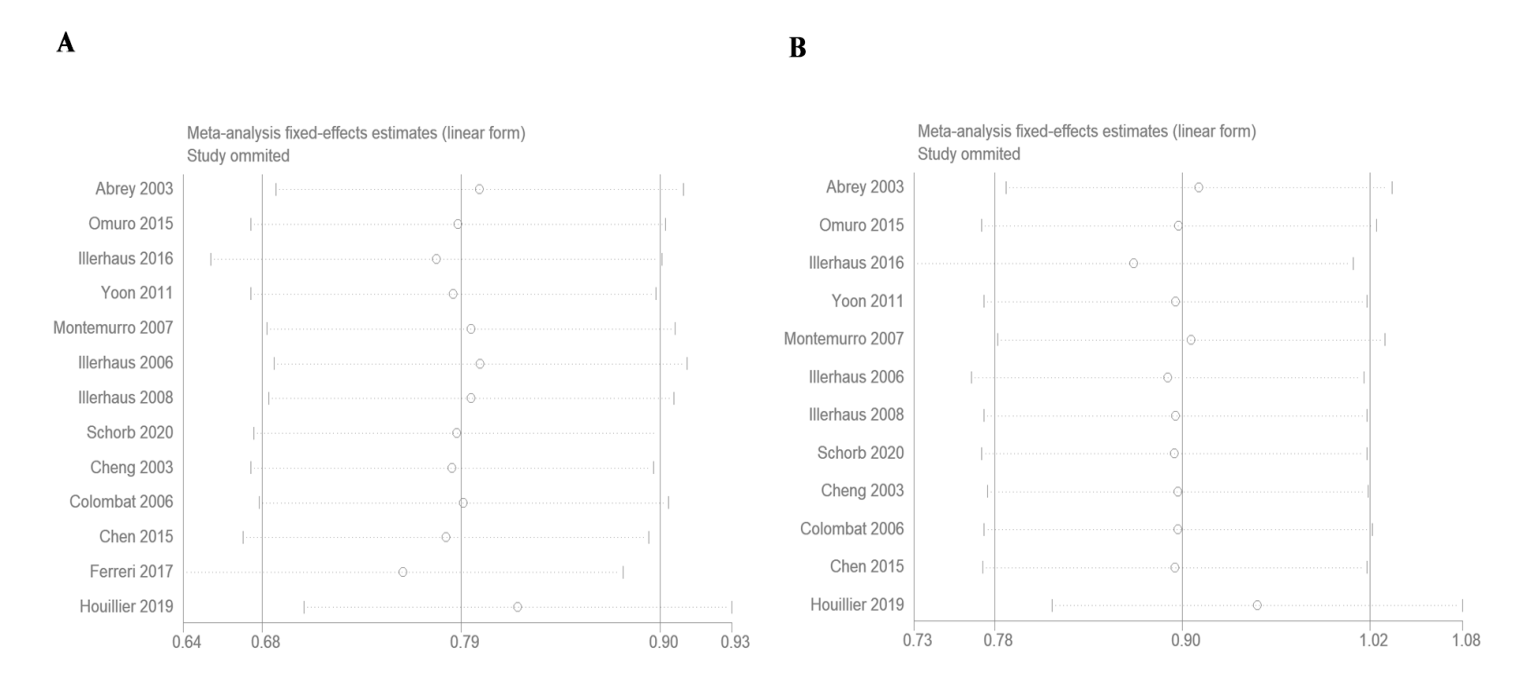


**Supplementary Figure 5.** The sensitivity analysis of the complete remission rate and overall response rate. (A) Complete remission rate. (B) Overall response rate.


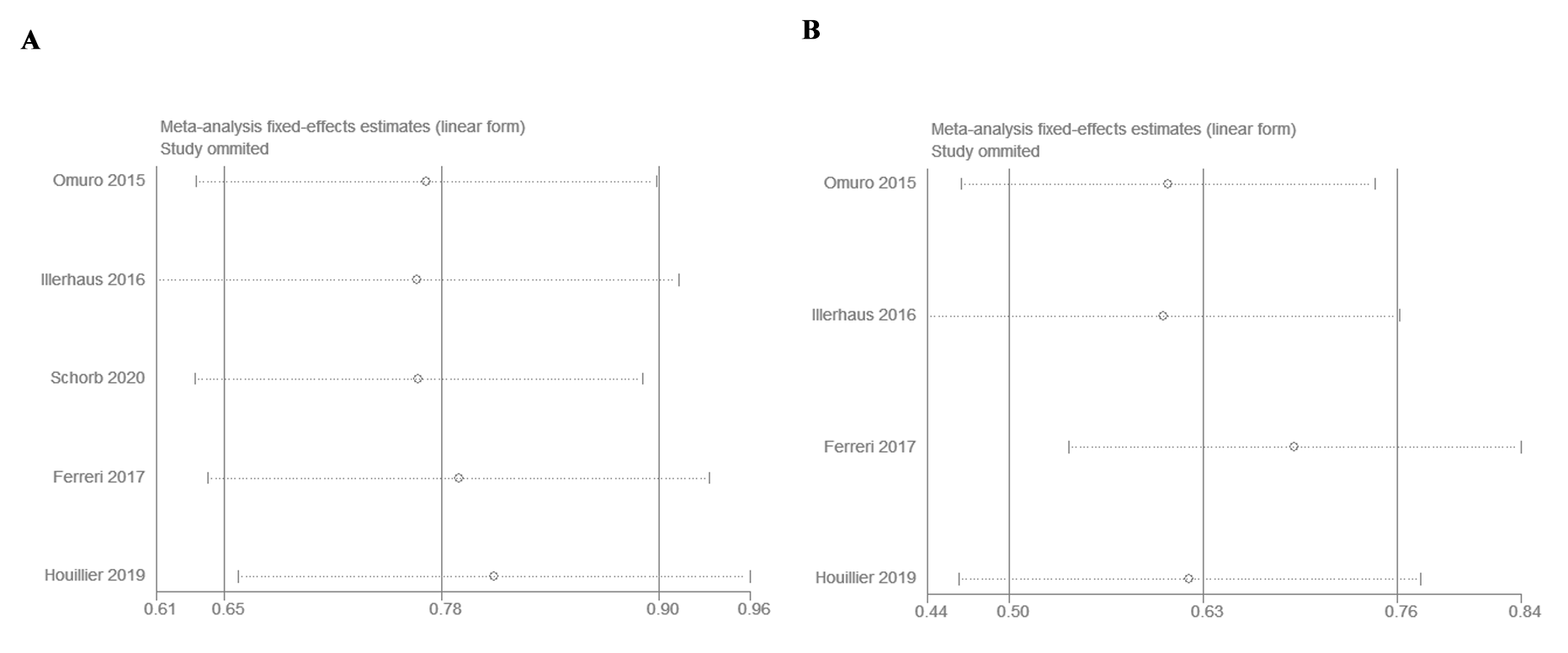


**Supplementary Figure 6.** The sensitivity analysis of the progression-free survival. (A) 1-year progression-free survival. (B) 5-year progression-free survival.


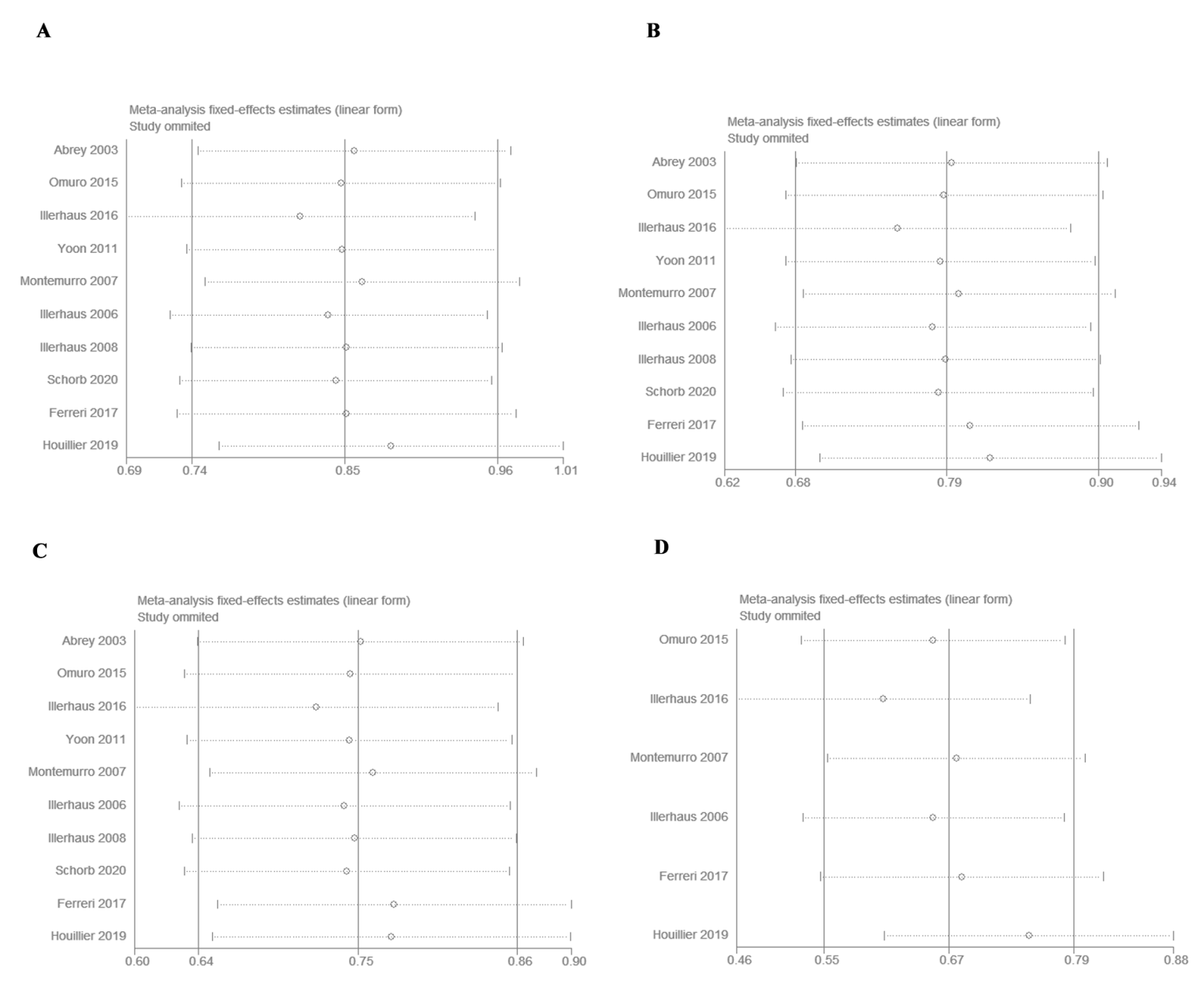


**Supplementary Figure 7.** The sensitivity analysis of the overall survival. (A) 1-year overall survival. (B) 2-year overall survival. (C) 3-year overall survival. (D) 5-year overall survival.


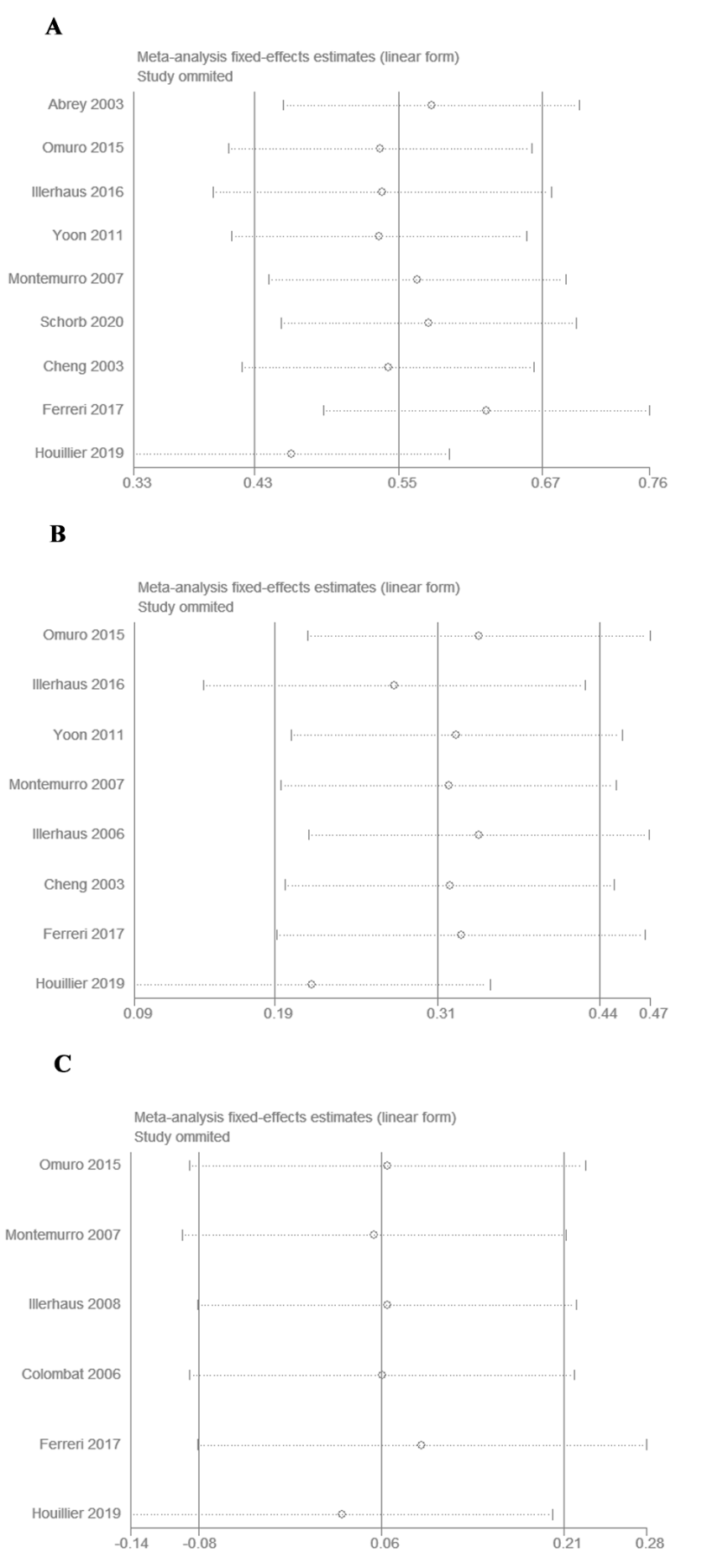


**Supplementary Figure 8.** The sensitivity analysis of the toxicity. (A) Febrile neutropenia or infections. (B) Mucositis. (C) Acute neurotoxicity/ Encephalopathy.


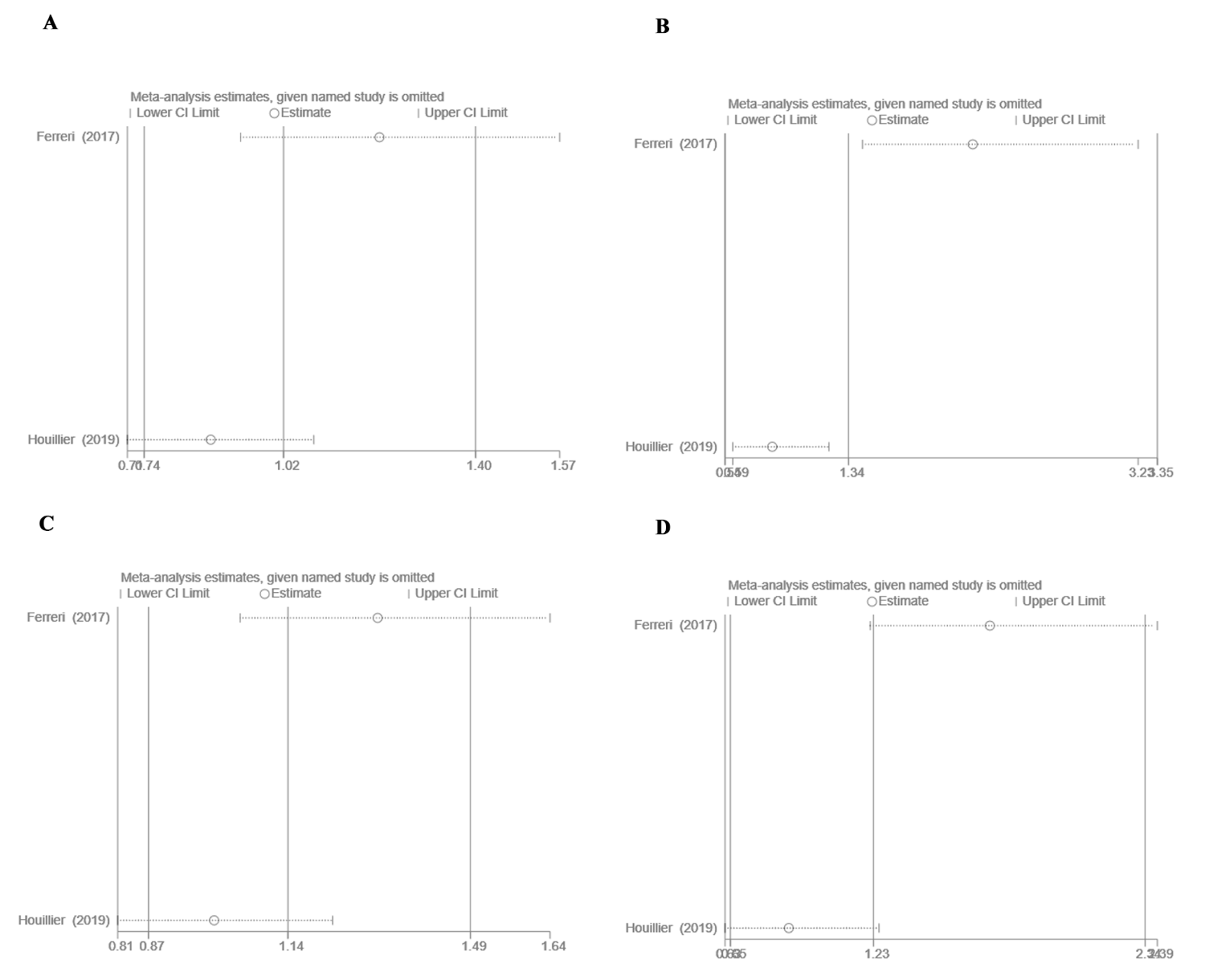


**Supplementary Figure 9.** The sensitivity analysis of the progression-free survival (PFS) for treatment with the ASCT group vs. WBRT group. (A) 2-year PFS in the intention-to-treat analyses. (B) 5-year PFS in the intention-to-treat analyses. (C) 2-year PFS in the per-protocol analyses. (D) 5-year PFS in the per-protocol analyses.
